# Diagnostic accuracy of PSA derivatives for prostate cancer in patients with low prostate-specific antigen levels

**DOI:** 10.3389/fonc.2025.1602134

**Published:** 2025-11-19

**Authors:** Aihemaiti Kadeer, Aerken Maolake, Aihemaiti Aimaier, Mukadaisi Abuduwaili, Zecheng Ni, Jiuzhi Li

**Affiliations:** 1 Department of Urology, People’s Hospital of Xinjiang Uygur Autonomous Region, Urumqi, China; 2 Department of Cancer Genetics, Roswell Park Comprehensive Cancer Center, Buffalo, NY, United States

**Keywords:** prostate cancer, TPSA, t/fPSA, PSAD, PSA-AV

## Abstract

Prostate-specific antigen (PSA) has long been used to screen for prostate cancer (PCa), yet its low diagnostic sensitivity in the so-called PSA “gray zone” often results in overdiagnosis or missed diagnoses. This study aimed to identify reliable diagnostic markers for PCa in patients with serum PSA levels ≤10 ng/mL by comparing various PSA derivatives. Clinical data from 60 patients (PSA ≤10 ng/mL) treated between 2013 and 2023 were retrospectively analyzed. Prostate volume was measured via suprapubic ultrasonography. Receiver operating characteristic (ROC) curves were used to evaluate the diagnostic performance of total PSA (tPSA), free PSA (fPSA), the free-to-total PSA ratio (f/tPSA), PSA density (PSAD), and the PSA-age volume index (PSA-AV). Area under the curve (AUC) values for tPSA, fPSA, f/tPSA, PSAD, and PSA-AV were 0.8301, 0.6830, 0.7225, 0.9318, and 0.9103, respectively. fPSA demonstrated the lowest diagnostic accuracy. tPSA showed moderate performance. Both PSAD and PSA-AV outperformed tPSA and f/tPSA, with positive predictive values of 89.47% and 74.07% and negative predictive values of 87.80% and 93.94%, respectively. PSAD demonstrated higher specificity (94.74%), while PSA-AV showed higher sensitivity (90.91%). PSAD appears to be a superior noninvasive diagnostic marker, while PSA-AV holds promise as an effective screening tool in patients with PSA ≤10 ng/mL. Given the small sample size, these findings should be regarded as preliminary and hypothesis-generating, pending validation in larger multicenter cohorts.

## Introduction

Prostate cancer (PCa) is the second most prevalent malignancy among men globally and a leading contributor to cancer-related morbidity and mortality (1). Early detection is critical for improving treatment outcomes; however, the current screening approach—primarily based on serum prostate-specific antigen (PSA) levels and digital rectal examination (DRE)—has limitations, particularly in the PSA “gray zone” (4–10 ng/mL). Within this range, only 25%–30% of patients are ultimately diagnosed with PCa, which leads to a high rate of unnecessary prostate biopsies (2).

The total PSA (tPSA) test lacks specificity, as PSA can also be elevated in benign conditions such as benign prostatic hyperplasia (BPH) or prostatitis. To improve diagnostic accuracy and minimize overtreatment, various PSA derivatives have been proposed, including free PSA (fPSA), the free-to-total PSA ratio (f/tPSA), PSA density (PSAD), and, more recently, the PSA-age volume index (PSA-AV). While some studies support the diagnostic value of f/tPSA and PSAD (3–5), others question their added benefit (6–8).

PSAD adjusts PSA values for prostate volume, thereby accounting for PSA elevation due to benign enlargement, while PSA-AV integrates age, PSA level, and prostate volume into a single composite measure. These indices may offer improved discrimination between malignant and benign conditions and help stratify patients for further diagnostic workup. Despite the potential of these markers, their clinical adoption has been limited by variable study results and a lack of consensus on optimal cutoff values.

This study aims to assess the comparative diagnostic performance of tPSA, fPSA, f/tPSA, PSAD, and PSA-AV in patients with serum PSA levels ≤10 ng/mL. By evaluating their ability to distinguish PCa from BPH, we seek to propose a more precise and practical approach to early diagnosis and triage in this diagnostically challenging population.

## Method and materials

### Patient selection

This retrospective analysis included 60 male patients with serum PSA levels ≤10 ng/mL, treated at the Urology Department of the People’s Hospital of Xinjiang Uygur Autonomous Region between May 2013 and May 2023. None of the patients had received prior treatment. Blood samples were collected on the second day of admission, and PSA levels were measured by the hospital laboratory. Prostate volume was assessed using suprapubic ultrasonography, and prostate biopsies were performed under ultrasound guidance. Based on pathological findings, patients were categorized into two groups: BPH (*n* = 38) and PCa (*n* = 22).

### Statistical analysis

GraphPad Prism (GraphPad Software, San Diego, CA, USA) was used for statistical evaluation. Receiver operating characteristic (ROC) curve analysis assessed the discriminative power of each PSA derivative. Optimal cutoff values were determined using the Youden index. Positive predictive value (PPV) and negative predictive value (NPV) were calculated using standard definitions. For each area under the curve (AUC) value, 95% confidence intervals (CIs) were computed to assess robustness. A *p*-value <0.05 was considered statistically significant.

## Results

### Patient demographics and baseline characteristics

A total of 60 male patients were included, comprising 38 patients diagnosed with BPH and 22 patients diagnosed with PCa, all with serum PSA levels ≤10 ng/mL. The mean age of patients with PCa was significantly higher than that of patients with BPH (70.32 ± 1.95 years vs. 64.24 ± 1.08 years; *p* = 0.0044). Prostate volume was significantly smaller in patients with PCa (38.69 ± 4.70 mL) compared to the BPH group (69.15 ± 6.95 mL; *p* = 0.0030), which supports the consideration of prostate size in PSA interpretation (**Table 1**).

**Table 1:** Comparison of laboratory and ultrasound parameters between patients with BPH and those with PCa (mean ± SE).

Parameter	BPH (*n* = 38)	PCa (*n* = 22)	*p*-value
Age (years)	64.237 ± 1.084	70.318 ± 1.951	0.0044
tPSA (ng/mL)	3.759 ± 0.465	7.410 ± 0.353	<0.0001
fPSA (ng/mL)	0.960 ± 0.116	1.412 ± 0.154	0.0223
f/tPSA ratio	0.270 ± 0.018	0.191 ± 0.017	0.0052
Prostate volume (mL)	69.148 ± 6.951	38.688 ± 4.700	0.0030
PSAD (ng/mL/cm^3^)	0.067 ± 0.010	0.235 ± 0.024	<0.0001
PSA-AV index	2,756.190 ± 791.971	379.994 ± 51.292	0.0267

Regarding serum markers, tPSA was significantly elevated in the PCa group (7.41 ± 0.35 ng/mL) compared to the BPH group (3.76 ± 0.47 ng/mL; *p* < 0.0001). Similarly, fPSA values were significantly higher in patients with PCa (1.41 ± 0.15 ng/mL) than in patients with BPH (0.96 ± 0.12 ng/mL; *p* = 0.0223). However, the f/tPSA was significantly lower in patients with PCa (0.191 ± 0.017) than in patients with BPH (0.270 ± 0.018; *p* = 0.0052), aligning with the known inverse relationship between f/tPSA and cancer risk. Importantly, both PSAD and PSA-AV showed statistically significant differences between groups. PSAD was markedly elevated in patients with PCa (0.235 ± 0.024 ng/mL/cm³) compared to patients with BPH (0.067 ± 0.010; *p* < 0.0001), while PSA-AV was significantly lower in patients with PCa (379.99 ± 51.29) than in patients with BPH (2756.19 ± 791.97; *p* = 0.0267).

### Diagnostic performance

ROC curve analysis demonstrated that PSAD had the highest diagnostic accuracy (AUC = 0.9318), followed by PSA-AV (AUC = 0.9103), tPSA (AUC = 0.8301), f/tPSA (AUC = 0.7225), and fPSA (AUC = 0.6830) (**Table 2**). PSAD showed a sensitivity of 77.27% and a specificity of 94.74%, while PSA-AV exhibited a higher sensitivity of 90.91% but a slightly lower specificity of 81.58% (**Table 3**). The PPV and NPV for PSAD were 89.47% and 87.80%, respectively. In contrast, PSA-AV demonstrated a PPV of 74.07% and an NPV of 93.94%.

**Table 2:** ROC curve analysis for PSA derivatives in PCa detection (PSA < 10 ng/mL).

Variable	AUC	Cutoff	Sensitivity	Specificity	95% confidence interval	*p*-value
tPSA	0.8301	4.0500	100%	60.53%	0.729457–0.930830	<0.0001
fPSA	0.6830	0.7950	90.91%	52.63%	0.548988–0.817040	0.0789
f/t PSA	0.7225	0.1990	63.64%	76.32%	0.586965–0.858011	0.0043
PSAD	0.9318	0.1772	77.27%	94.74%	0.871814–0.991823	<0.0001
PSA-AV	0.9103	555.1510	90.91%	81.58%	0.835121–0.985453	<0.0001

AUC, area under the curve.

**Table 3:** Positive and negative predictive values for PSAD and PSA-AV.

Variable	AUC	PPV (%)	NPV (%)	Sensitivity (%)	Specificity (%)
PSAD	0.9318	89.47%	87.80%	77.27%	94.74%
PSA-AV	0.9103	74.07%	93.94%	90.91%	81.58%

PPV, positive predictive value; NPV, negative predictive value.

Overall, PSAD provided the greatest diagnostic precision, combining high specificity and PPV, making it more suitable for confirming a PCa diagnosis. Meanwhile, PSA-AV offered superior sensitivity and NPV, supporting its potential role as a screening tool to help rule out malignancy in patients with PSA levels ≤10 ng/mL.

## Discussion

### Interpretation of results

This study aimed to evaluate the utility of PSA derivatives in diagnosing PCa among men with low to intermediate PSA levels (≤10 ng/mL), a population that presents a diagnostic challenge due to the overlap of values between benign and malignant prostate conditions.

Our results confirmed that tPSA alone is moderately effective at distinguishing PCa from BPH (AUC = 0.8301), but its specificity was limited (60.53%), reinforcing concerns about false positives and unnecessary biopsies. fPSA, although elevated in patients with PCa in absolute terms, showed poor diagnostic discrimination (AUC = 0.683), and the f/tPSA ratio offered only marginal improvement (AUC = 0.7225), consistent with prior findings that suggest limited additional value in the gray zone (3–6). These concerns echo earlier reports emphasizing the limitations of PSA-based screening and the need for refined diagnostic thresholds (10–12).

### Superiority of PSAD and PSA-AV

The standout markers in our analysis were PSAD and PSA-AV. PSAD had the highest AUC (0.9318), confirming its reliability in differentiating malignant from benign conditions. A cutoff of 0.177 ng/mL/cm³ yielded a sensitivity of 77.27% and a specificity of 94.74%, indicating that PSAD is highly specific and reduces unnecessary biopsies. This is comparable to the study by Khalid et al. (9), which reported a similar diagnostic advantage for PSAD at a slightly lower cutoff (0.155 ng/mL/cm³). PSA-AV, a newer index that incorporates PSA level, patient age, and prostate volume, performed nearly as well (AUC = 0.9103). Notably, it demonstrated superior sensitivity (90.91%) and NPV (93.94%), making it particularly valuable in a screening context where minimizing false negatives is paramount. These findings support the concept that incorporating prostate volume and age into PSA interpretation refines diagnostic precision. The use of volumetric indices like PSAD and PSA-AV effectively mitigates the influence of benign prostatic enlargement on PSA levels, which is especially relevant in older men.

### Clinical implications

From a clinical standpoint, the combined use of PSA-AV for screening and PSAD for diagnosis may offer a more nuanced approach to patient triage. PSA-AV, because of its high sensitivity, is particularly effective in population-level screening and can help rule out PCa with a high degree of confidence in low-risk individuals. In contrast, PSAD, which has higher specificity, is better suited for diagnostic confirmation in cases of elevated PSA, as it helps reduce the likelihood of unnecessary biopsies. This integrated approach can enhance the overall efficiency of PCa detection while minimizing the risk of overdiagnosis and overtreatment. Integrating these metrics with DRE and magnetic resonance imaging (MRI) where available could form a robust pre-biopsy risk assessment framework, especially in settings with limited access to advanced imaging. From a clinical perspective, PSAD and PSA-AV may be integrated into current diagnostic algorithms alongside multiparametric MRI or other emerging biomarkers to improve risk stratification and guide biopsy decisions in patients within the PSA gray zone. In addition, standardization of prostate volume assessment—preferably through MRI-based or automated ultrasound measurements—will be critical for enhancing reproducibility and enabling cross-center comparison in future studies.

### Study limitations

Several limitations should be acknowledged. The retrospective, single-center design and limited sample size constrain the generalizability and statistical power of this study, and therefore, the findings should be regarded as exploratory. In addition, the lack of an external validation cohort limits the robustness of the proposed cutoff values. Prostate volume was assessed using suprapubic ultrasonography, which is less accurate than transrectal or MRI-based methods and may have introduced operator-dependent variability affecting PSAD and PSA-AV calculations. Future multicenter, prospective studies with larger cohorts and standardized imaging modalities are warranted to validate and strengthen these preliminary findings.

### Summary

**Figure 1:**
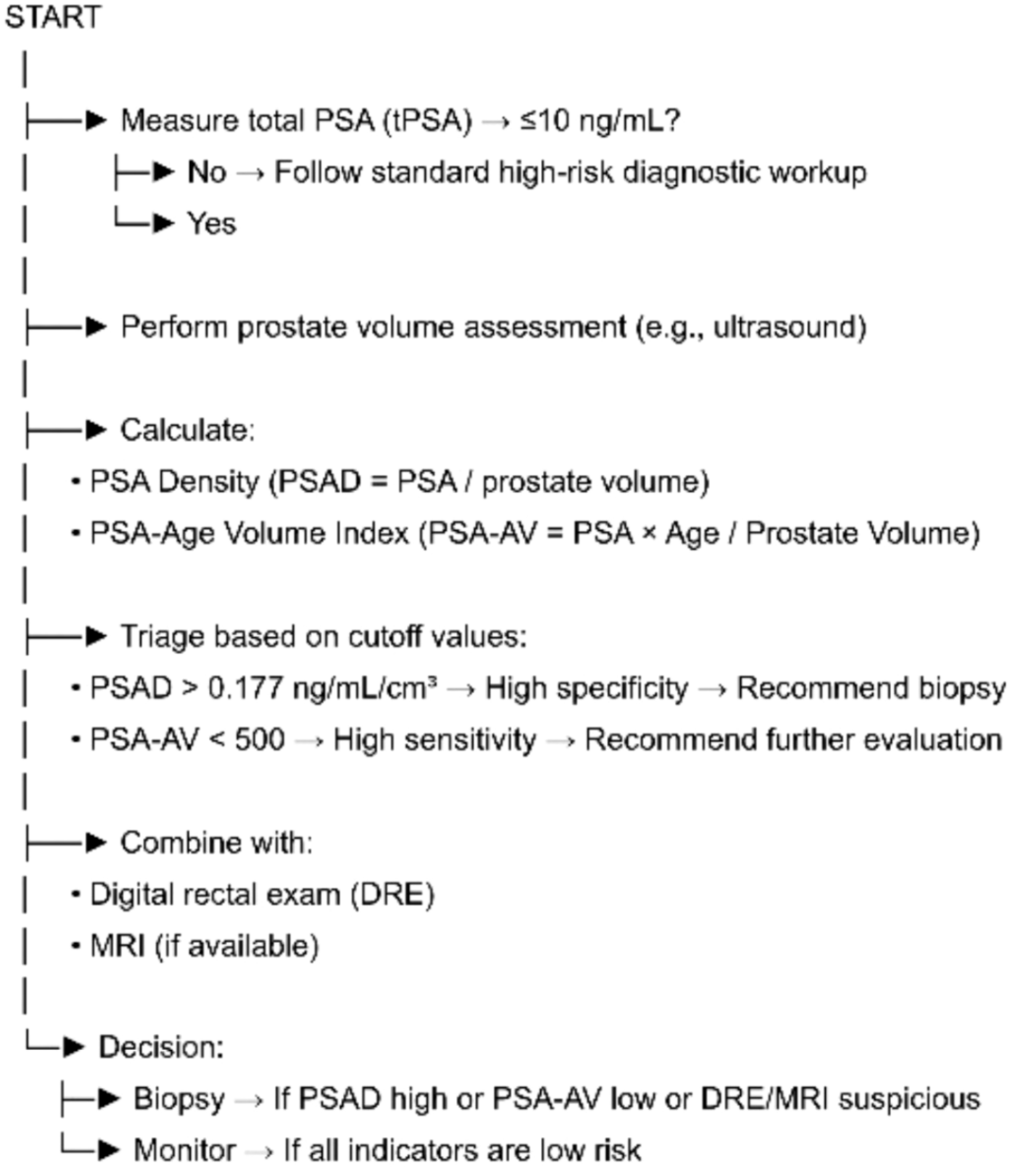
Flowchart outlining a triage approach for prostate cancer evaluation in men with total PSA ≤ 10 ng/mL. After confirming PSA levels, prostate volume is measured to calculate PSA density (PSAD) and PSA-age volume index (PSA-AV). PSAD > 0.177 ng/mL/cm³ indicates high specificity for clinically significant prostate cancer and suggests the need for biopsy. PSA-AV < 500 offers high sensitivity and supports further evaluation. These indices are integrated with DRE and MRI findings, if available, to guide the final clinical decision: biopsy or active monitoring.

This study reinforces the limited diagnostic value of tPSA and fPSA alone in men with PSA levels ≤10 ng/mL, while highlighting the superior diagnostic and screening potential of PSAD and PSA-AV. PSAD demonstrates the highest diagnostic accuracy, driven by its strong specificity and PPV, making it well-suited for confirming PCa in individuals with elevated PSA. In contrast, PSA-AV proves more effective as a screening tool due to its high sensitivity and NPV, allowing for better identification of low-risk individuals. Integrating these indices into clinical workflows may enhance early detection of PCa while reducing unnecessary interventions and associated harms (**Figure 1**). Although these results are encouraging, they should be regarded as preliminary, and both PSAD and PSA-AV require validation in larger, independent cohorts before clinical implementation.

## Data Availability

The original contributions presented in the study are included in the article/supplementary material. Further inquiries can be directed to the corresponding author.
